# Supplementary material for: G4-Ligand-Conjugated Oligonucleotides Mediate Selective Binding and Stabilization of Individual G4 DNA Structures
Source: J Am Chem Soc. 2024 Mar 2;146(10):6926–35. doi: 10.1021/jacs.3c14408 (PMC10941181; doi:10.1021/jacs.3c14408)
Supplement: Supplementary file 1 — ja3c14408_si_001.pdf [file ja3c14408_si_001.pdf]

G4-Ligand Conjugated Oligonucleotides Mediate Selective Binding and Stabilization of Individual G4 DNA Structures

Andreas Berner[#b], Rabindra Nath Das[#a], Naresh Bhuma[#a], Justyna Golebiewska[a], Alva Abrahamsson[a], Måns Andréasson[a], Namrata Chaudhari[b], Mara Doimo[b], Partha Pratim Bose[c], Karam Chand[a], Roger Strömberg[c], Sjoerd Wanrooij*[b], Erik Chorell*[a]

[#] Equally contributing first authors, *Equally contributing corresponding authors

[a] Department of Chemistry, Umeå University, 901 87, Umeå, Sweden

[b] Department of Medical Biochemistry and Biophysics, Umeå University, Umeå 901 87, Sweden

[c] Department of Biosciences and Nutrition, Karolinska Institutet, Huddinge, Sweden.

Supporting Information

Table of Contents

Table S1. Oligonucleotides used in this study. **3**

Figure S1. .... **4**

Figure S2. .... **4**

Figure S3. .... **5**

Figure S4. .... **5**

Figure S5. .... **6**

Figure S6. .... **7**

Figure S7. .... **8**

Figure S8. .... **9**

Figure S9. .... **10**

Figure S10. .... **11**

General Experimental ***12***

Synthesis **12**

Table S2. .... **14**

NMR Spectra of Synthesised Compounds ***15***

Mass chromatograms of synthesised GL-Os ***18***

References ***24***

Table S1. Oligonucleotides used in this study.

Sequence name	Sequence
**Oligos/Oligo-Compounds**	
CO	5′-AGC ACT CTA GCT CTA-3'
O15	5′-TAG AGC TAG AGT GCT-3′
O15^{LNA}	5′-**TAG** AGC TAG AGT **GCT**-3′
GL-O15	G4-ligand(**6**)-5′-TAG AGC TAG AGT GCT-3′
GL-O15^{LNA}	G4-ligand(**6**)-5′-**TAG** AGC TAG AGT **GCT**-3′
GL-O15^{ALT}	G4-ligand(**6**)-5′-TCA CGT CCA ATT CAG-3′
GL-O16	G4-ligand(**6**)-5′-**A**TA GAG CTA GAG TGC T-3′
GL-O17	G4-ligand(**6**)-5′-**CA**T AGA GCT AGA GTG CT-3′
GL-O15 8T	G4-ligand(**6**)-5′-TAG AGC T**T**G AGT GCT-3′
GL-O15 8&11T	G4-ligand(**6**)-5′-TAG AGC T**T**G A**T**T GCT-3′
GL-O15 1 MM	G4-ligand(**6**)-5′-**A**AG AGC TAG AGT GCT-3′
GL-O15 2 MM	G4-ligand(**6**)-5′-T**T**G AGC TAG AGT GCT-3′
GL-O15 1,2 MM	G4-ligand(**6**)-5′-**AT**G AGC TAG AGT GCT-3′
GL-O15 14 MM	G4-ligand(**6**)-5′-TAG AGC TAG AGT G**T**T-3′
GL-O15 15 MM	G4-ligand(**6**)-5′-TAG AGC TAG AGT GC**C**-3′
GL-O15 14,15 MM	G4-ligand(**6**)-5′-TAG AGC TAG AGT G**TC**-3′
25nt-TET primer	5′-TET-ATA GGG GTA TGC CTA CTT CCA ACT C-3′
25nt-A647 primer	5′-A647-ATA GGG GTA TGC CTA CTT CCA ACT C-3′
15nt-TET primer	5′-TET-GCC TAC TTC CAA CTC-3′
O20-T(10)	5′-TTT TTT TTT TTA GAG CTA GAG TGC TGC GAG-3′
**Templates**	
c-MYC Pu24T	5'-TGA GGG TGG TGA GGG TGG GGA AGG-3′
R1-Pu24T G4	5′-GAG ATT AGC GAG AGA TGA GGG TGG TGA GGG TGG GGA AGG-3′
R2-Pu24T (**G4 DNA**)	5′-AGC ACT CTA GCT CTA TGA GGG TGG TGA GGG TGG GGA AGG -3′
R3- Pu24T G4	5′-GAG AGG GAG CGG CTG TGA GGG TGG TGA GGG TGG GGA AGG -3′
Cy5-Pu24T G4	5′-Cy5-AGC ACT CTA GCT CTA TGA GGG TGG TGA GGG TGG GGA AGG-3′
Pu24T G4 Complementary Primer Extension	5′-AGC ACT CTA GCT CTA TGA GGG TGG TGA GGG TGG GGA AGG CAC GTG AGT TGA GTG GAG TTG GAA GTA GGC ATA CCC CTA T-3′
Pu24T G4 Non-Complementary Primer Extension	5′-CTG AAT TGG ACG TGA TGA GGG TGG TGA GGG TGG GGA AGG CAC GTG AGT TGA GTG GAG TTG GAA GTA GGC ATA CCC CTA T-3′
HelB-G4-1 Complementary Primer Extension	5′-AGC ACT CTA GCT CTA TAG GGG AGG GCA TGT GGA AGG GGC GGG GCA CGT GAG TTG AGT GGA GTT GGA AGT AGG CAT ACC CCT AT-3′
c-Kit-G4 Complementary Primer Extension	5′-AGC ACT CTA GCT CTA TAG GGA GGG CGC TGG GAG GAG GGG CAC GTG AGT TGA GTG GAG TTG GAA GTA GGC ATA CCC CTA T-3′
Pu24T G4 20nt Complementary Primer Extension	5′-CTC GCA GCA CTC TAG CTC TAT GAG GGT GGT GAG GGT GGG GAA GGC ACT GAG TAT GGA GTT GGA AGT AGG C-3′

Changes to the sequence of GL-O15 are marked in green. Nucleotides changed to LNA are marked in yellow. G4 forming regions of the templates are marked in red. MM stands for MisMatch.

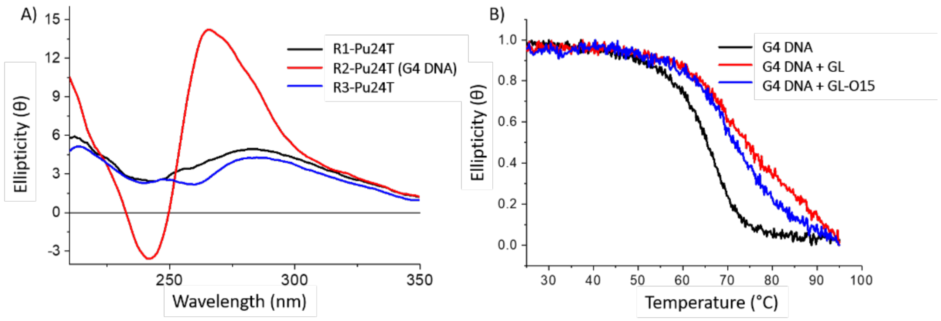

Figure S1. A) CD of *c-MYC* Pu24T G4 with different flanking sequences (R1-R3, see table S1). **B)** CD melting curves of *c-MYC* Pu24T with flanking sequence (called G4 DNA) (black) in presence of GL (red) and GL-O15 (blue).

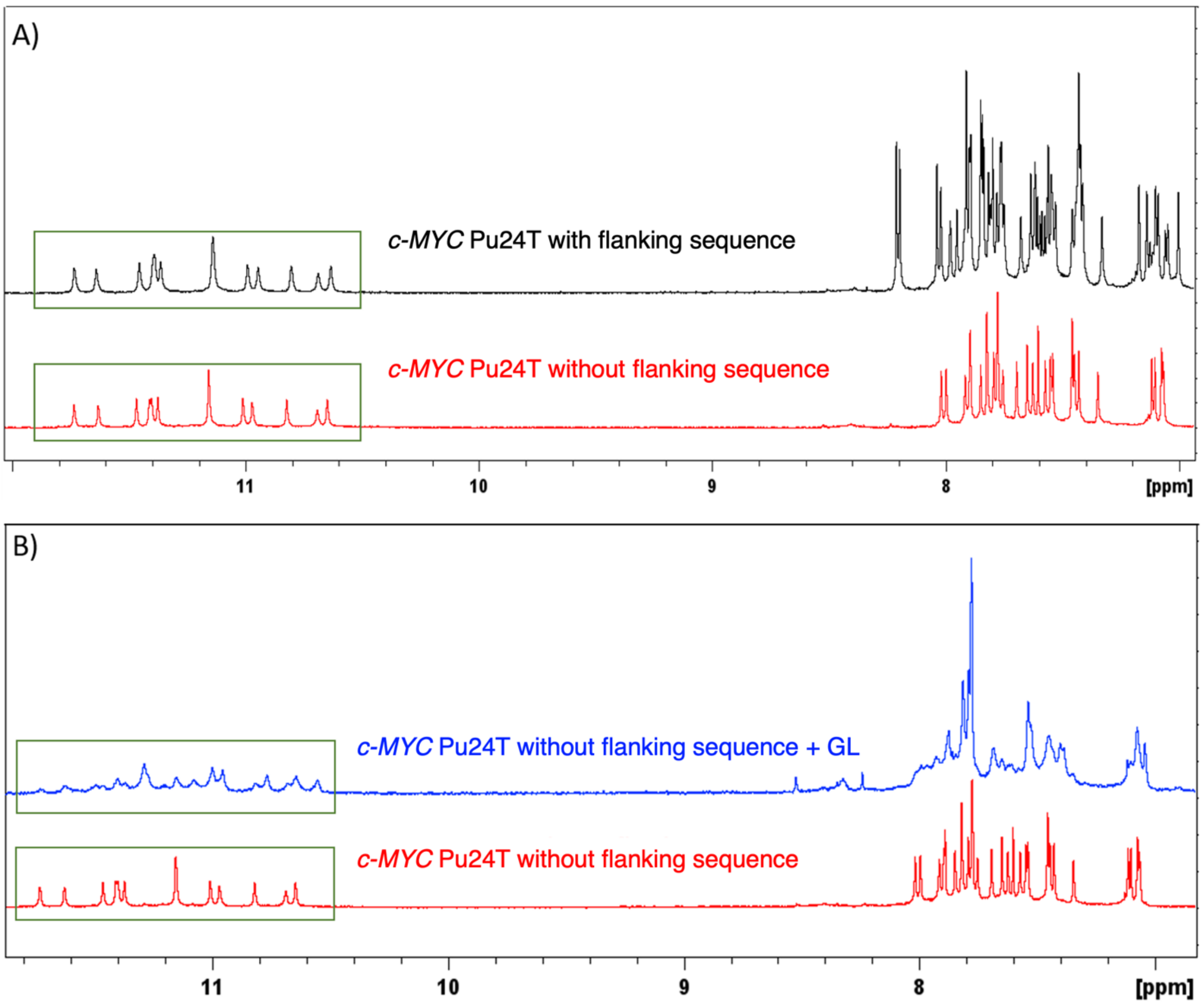

Figure S2. A) ^{1}H NMR Spectra of *c-MYC* Pu24T G4 DNA with the 15-nt flanking DNA sequence (black), and *c-MYC* Pu24T without the 15-nt flanking DNA sequence (red). **B)** ^{1}H NMR Spectra of *c-MYC* Pu24T G4 in presence (blue) and absence (red) of GL.

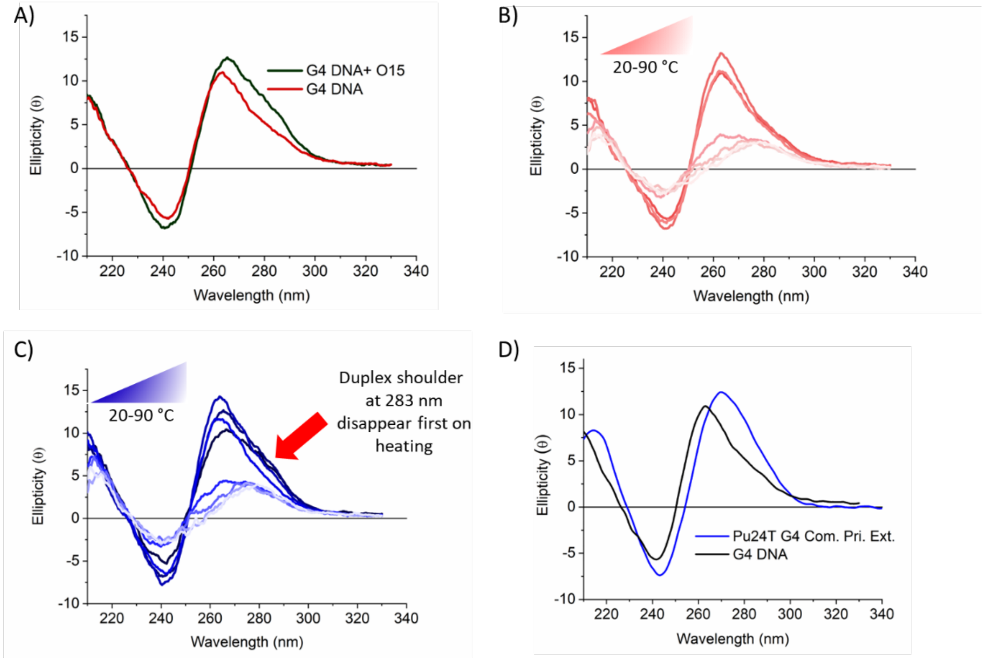

Figure S3. A) CD spectrum of G4 DNA (red) and in the presence of O15 (black), showing a shoulder (270-290 nm) signal that is indicative of double-stranded DNA formation between the G4 DNA and O15. **B)** CD spectra of Pu24T in <u>absence</u> of O15 at different temperatures. **C)** CD spectra of G4 DNA in <u>presence</u> of O15 at different temperatures. The shoulder at 283 nm correspond to dsDNA and this shoulder disappear first which suggest that dsDNA is denatured at lower temperatures compared to the G4 DNA structure. **D)** CD spectrum of G4 DNA (black) and Pu24T G4 with the same flanking sequences that were used in the DNA polymerase stop assay, called complementary primer extension (blue). The G4 with the flanking sequences used in the DNA polymerase stop assay show a positive peak at 269 nm and negative peak at 243 nm indicating a predominant parallel topology.

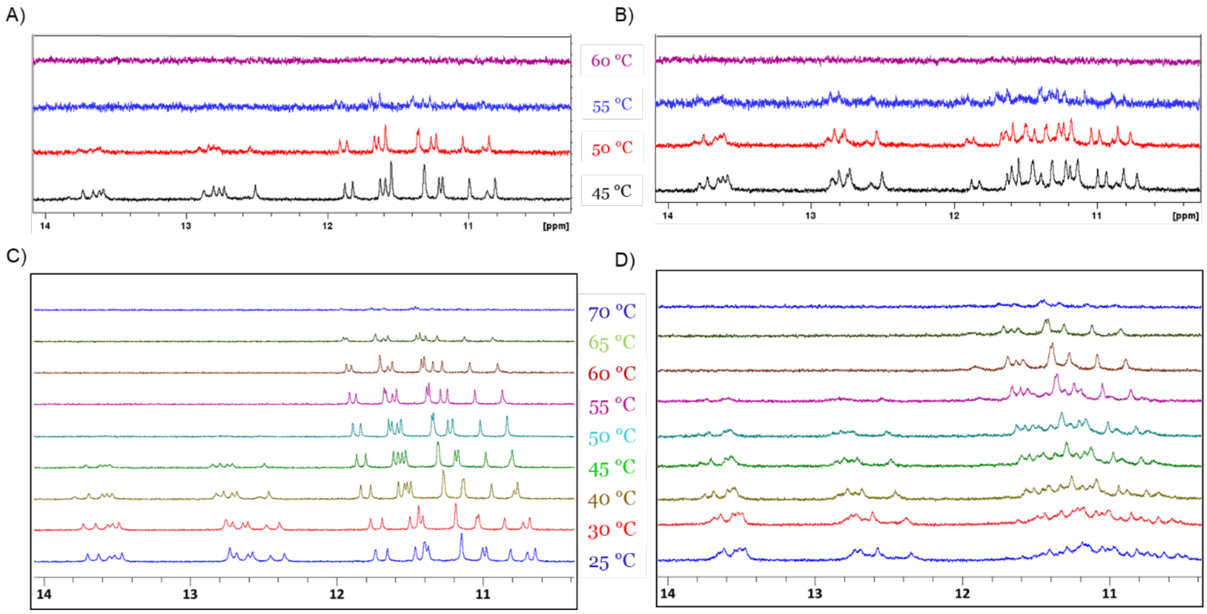

Figure S4. A) ^{1}H NMR Spectra of G4 DNA + O15 at different temperatures, **B**) G4 DNA + GL-O15 at different temperatures. Spectra for A) and B) was recorded in 500 µM KCl and 10 mM potassium phosphate buffer (pH = 7.4). **C)** ^{1}H NMR Spectra of G4 DNA + O15, **D**) G4 DNA + GL-O15 at different temperatures. Spectra for C) and D) was recorded in 3 mM KCl and 10 mM potassium phosphate buffer (pH = 7.4).

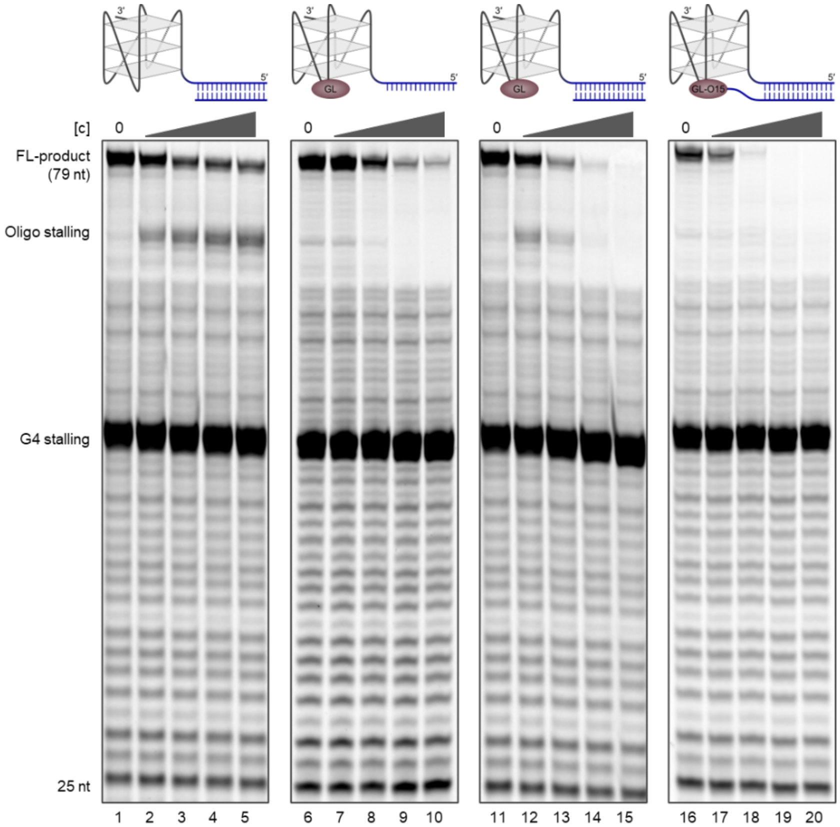

Figure S5. Polymerase-Stop-Assays in the presence of increasing concentrations (0.04, 0.2, 1 and 5 µM) of oligo alone, O15 (lanes 2-5), compound alone, GL (lanes 7-10), oligo + compound, O15 + GL (lanes 12-15) or oligo-linked compound, GL-O15 (lanes 17-20) on a DNA template that includes a 15-nts DNA sequence complementary to the oligo.

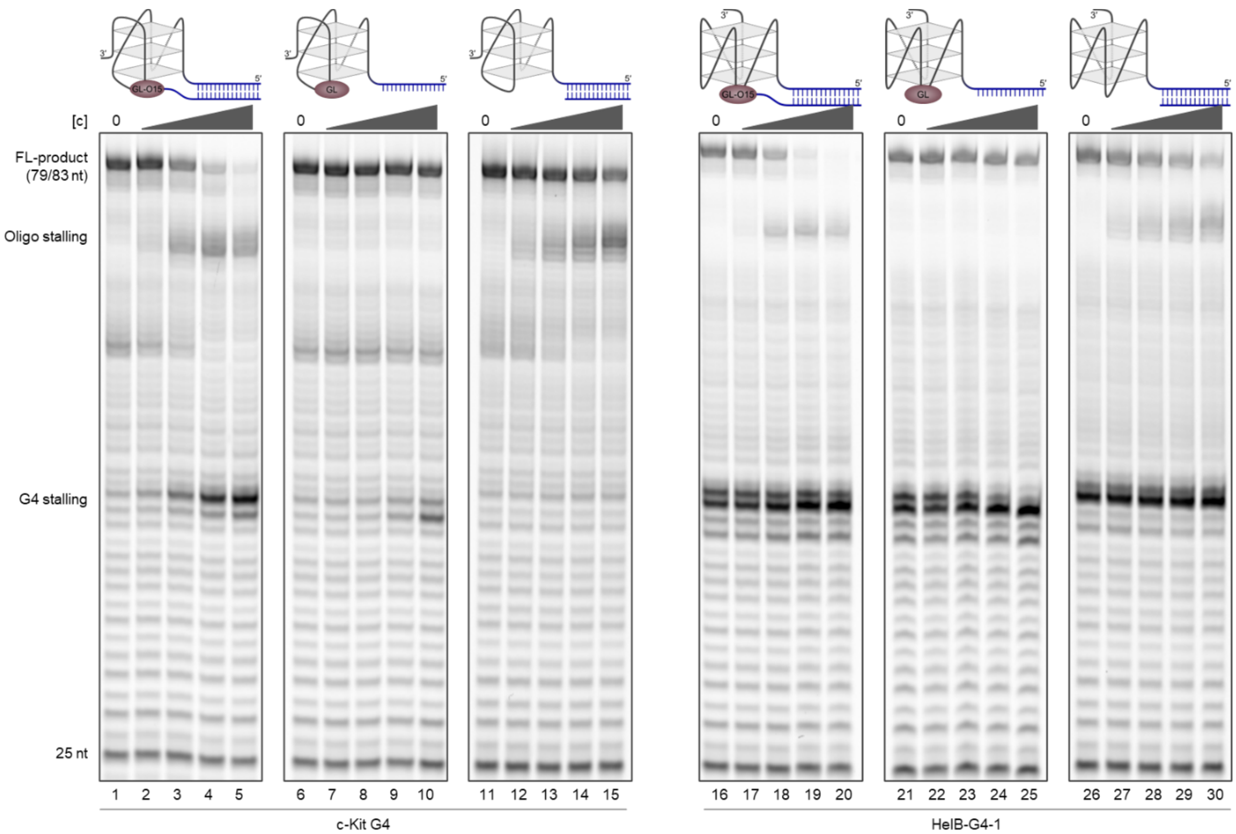

Figure S6. Polymerase-Stop-Assays with different G4 templates. *c-Kit 1* G4 (left) and *HelB* G4-1 (right) with the 15-nts complementary flanking sequence added, with increasing concentrations (0.04. 0.2, 1 and 5 µM) of GL-O15 (lanes 2-5 and 17-20), GL (lanes 7-10 and 22-25) or O15 (lanes 12-15 and 27-30).

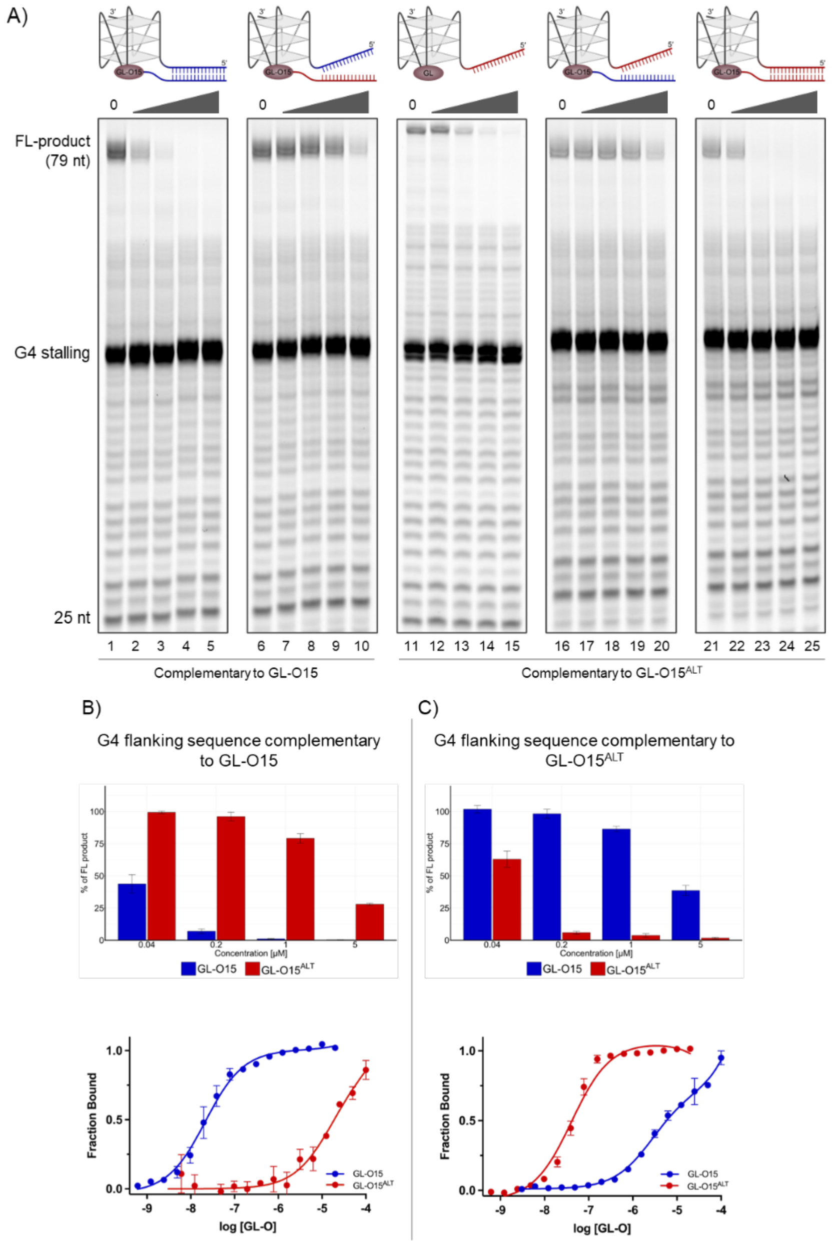

Figure S7. A) Polymerase-Stop-Assays with increasing concentrations (0.04. 0.2, 1 and 5 µM) of GL-O15 (lanes 2-5, 17-20), GL-O15^{ALT} (lanes 7-10, 22-25), or GL (lanes 12-15) on a DNA template with a flanking sequence matching GL-O15 (lanes 1-10) or a template with an alternative flanking sequence matching GL-O15^{ALT} (lanes 11-25). **B)** Top, quantification of data from A) with a G4 flanking sequence complementary to GL-O15 (lanes 2-5 and 7-10), percentage of full-length product compared to the control reaction without compound, mean and standard deviation of three individual experiments are shown. Bottom, dose-response curves obtained from MST analysis on G4 DNA with the flanking sequence complementary to GL-O15 after addition of GL-O15 and GL-O15^{ALT}. **C)** Top, quantification of data from A) with a G4 flanking sequence complementary to GL-O15^{ALT} (lanes 17-20 and 22-25), percentage of full-length product compared to the control reaction without compound, mean and standard deviation of three individual experiments are shown. Bottom, dose-response curves obtained from MST analysis on *c-MYC* Pu24T G4 DNA with the flanking sequence complementary to GL-O15^{ALT} after addition of GL-O15 and GL-O15^{ALT}.

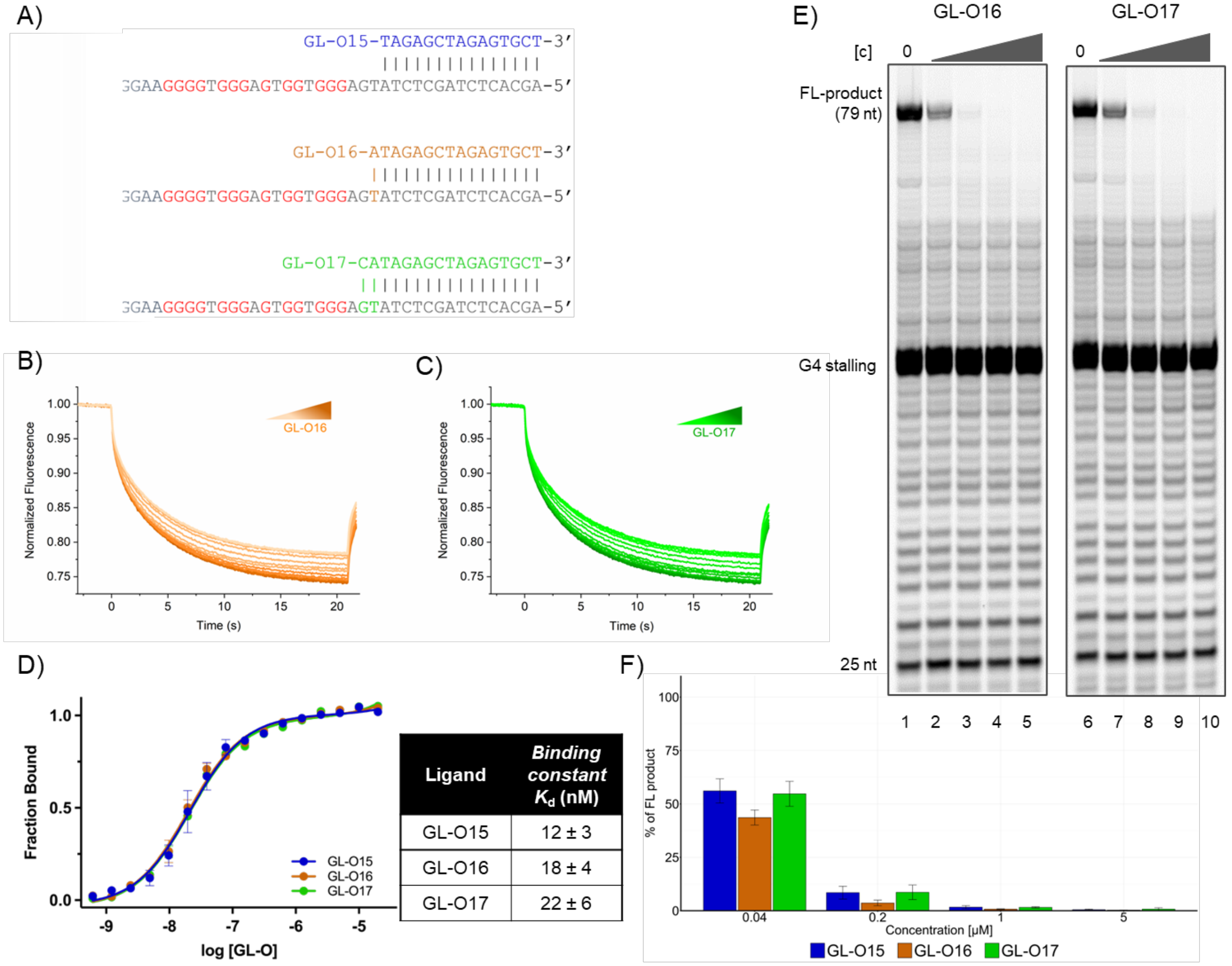

Ligand	*Binding constant* K_d (nM)
GL-O15	12 ± 3
GL-O16	18 ± 4
GL-O17	22 ± 6

Figure S8. A) Schematic representation of the different oligonucleotides added to the G4 stabilizing compounds. The 15-nucleotide DNA template binding sequence in GL-O15 is extended by one (GL-O16) or two (GL-O17) nucleotides towards the G4 forming sequence of the template. **B)** MST traces from titration of GL-O16 to Pu24T. **C)** MST traces from titration of GL-O17 to Pu24T. **D)** Dose-response curves from MST analysis of GL-O15, GL-O16, and GL-O17 with G4 DNA. Error bars correspond to SD of two independent measurements. Table of K_d values obtained from MST analysis. **E)** Polymerase-Stop-Assays was performed with increasing concentrations (0.04. 0.2, 1 and 5 μM) of GL-O16 (lanes 2-5) and GL-OL17 (lanes 7-10), on a template with the sequence complementary to the oligo DNA sequence. **F)** Quantification of E, percentage of full-length product compared to the control reaction without compound, mean and standard deviation of three individual experiments are shown.

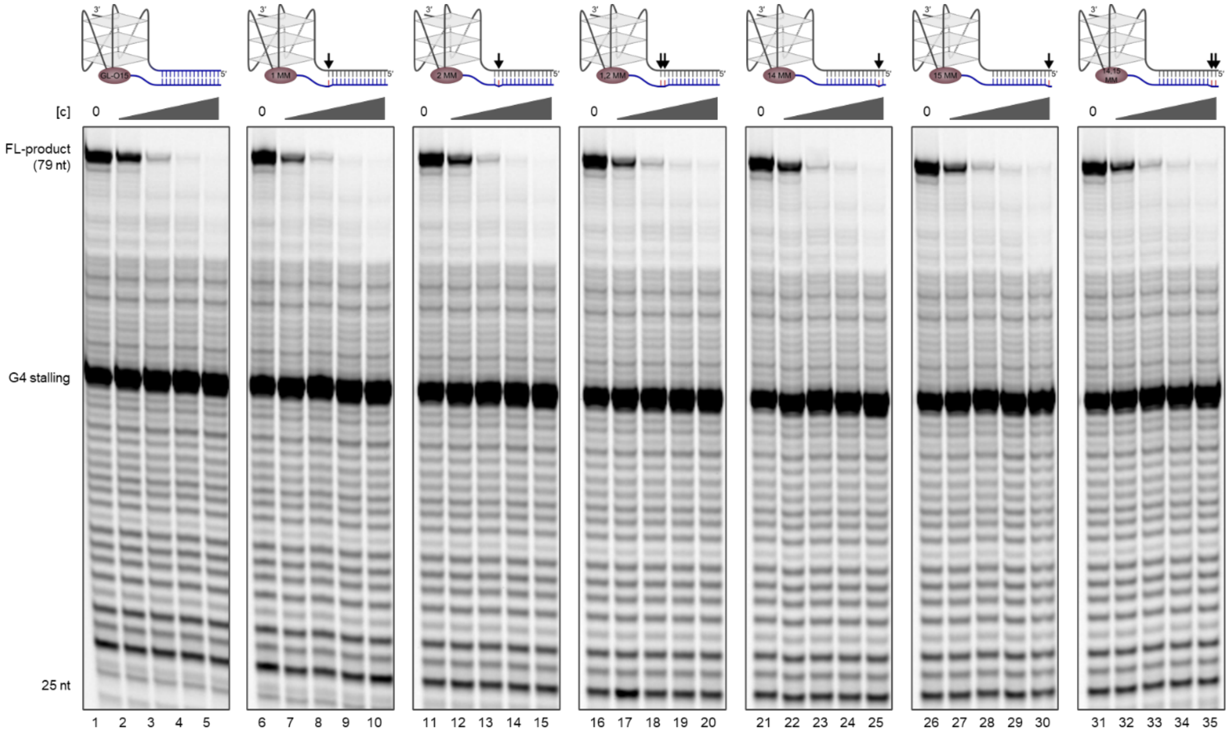

Figure S9. Polymerase-Stop-Assays were performed with increasing concentrations (0.04. 0.2, 1 and 5 µM) of GL-O15 (lanes 2-5), GL-O15 1 MM (lanes 7-10), GL-O15 2 MM (lanes 12-15, GL-O15 1,2 MM (lanes 17-20), GL-O15 14 MM (lanes 22-25), GL-O15 15 MM (lanes 27-30) and GL-O15 14,15 MM (lanes 32-25).

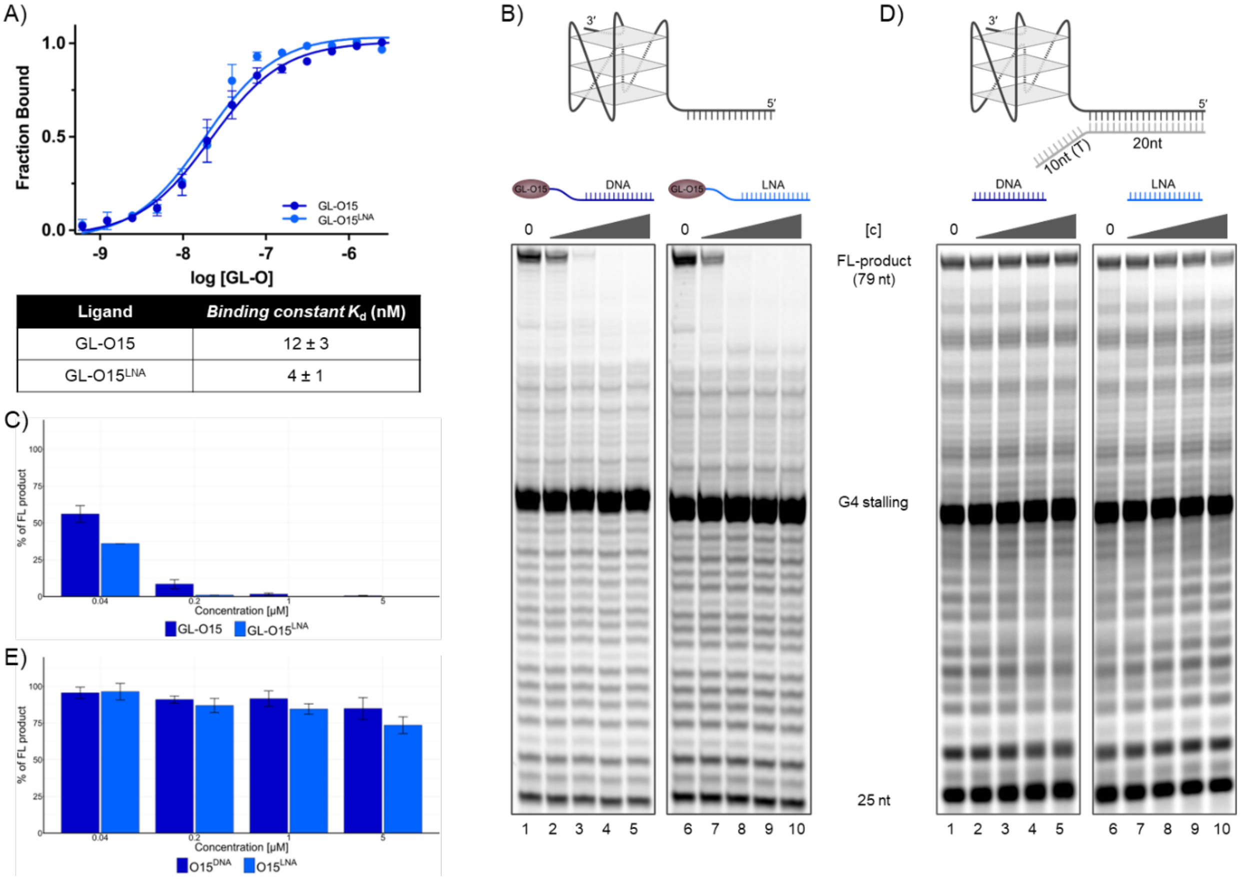

Figure S10. A) Dose-response curves from MST analysis of GL-O15 and GL-O15^{LNA} with G4 DNA. Error bars correspond to SD of two independent measurements. Table of K_d values obtained from MST analysis. **B)** Polymerase-Stop-Assays was performed with increasing concentrations (0.04. 0.2, 1 and 5 µM) of GL-O15^{DNA} (lanes 2-5) and GL-O15^{LNA} (lanes 7-10), on a template with the sequence complementary to the oligo DNA sequence. **C)** Quantification of B, percentage of full-length product compared to the control reaction without compound, mean and standard deviation of three individual experiments are shown. **D)** Polymerase-Stop-Assays with increasing concentrations (0.04. 0.2, 1 and 5 µM) of O15^{DNA} (lanes 2-5) and O15^{LNA} (lanes 7-10), on a template with the extended flanking sequence with the complementary oligo containing a T(10)-stretch annealed. **E)** Quantification of D, percentage of full-length product compared to the control reaction without compound, mean and standard deviation of three individual experiments are shown.

General Experimental

All reagents and solvents were purchased from commercial suppliers unless stated otherwise. TLC was performed on aluminium backed silica gel plates (median pore size 60 Å, fluorescent indicator 254 nm) and detected with UV light. Flash column chromatography was performed using silica gel with an average particle diameter of 50 µm (range 40−65 µm, pore diameter 53 Å), eluents are given in brackets. DMF were dried in a solvent drying system (DMF drying agent: activated molecular sieves, also equipped with an isocyanate scrubber) and were collected fresh prior to every reaction. ^{1}H and ^{13}C NMR spectra were recorded on a Bruker 400 MHz or 600 MHz spectrometer at 298 K, calibrated by using the residual peak of the solvents as the internal standard ($CDCl_3$: δ (ppm) H = 7.26; δ (ppm) C = 77.16. DMSO-d_6: δ (ppm) H = 2.50; δ (ppm) C = 39.50. Acetone-d_6: δ (ppm) H = 2.05; δ (ppm) C = 29.84, 206.26. CD_3OD: δ (ppm) H = 3.31; δ (ppm) C = 49.00). LC-MS was performed on an Agilent 6150 Series Quadrupole LC/MS system. HRMS was performed by using an Agilent 1290 binary LC System connected to an Agilent 6230 Accurate-Mass TOF LC/MS (ESI+); calibrated with Agilent G1969-85001 ESTOF Reference Mix containing ammonium trifluoroacetate, purine and hexakis (1H, 1H, 3H tetrafluoropropoxy) phosphazine in 90:10 CH_3CN:H_2O.

All chemical reagents and solvents including N, N-diisopropylethylamine (DIPEA), ethylenediamine tetraacetate (EDTA), triethylamine (TEA), acetic acid, acetonitrile (HPLC grade), methanol and dimethyl sulfoxide (DMSO), were purchased from *Sigma Aldrich*, and 1,1,1,3,3,3-hexafluoro-2-propanol (HFP) from *Fluorochem*. All the purchased compounds were used without further purification. The oligonucleotides were purchased from IDT (*Integrated DNA Technologies,* Sweden) and *FutureSynthesis* (Poland). Water used for reactions and buffers were in Milli-Q purity.

Reverse phase (RP) HPLC was carried out on a Hitachi HPLC system using Clarity 5 µm Oligo RP LC, Fully Porous Organo-silica C18 (250 x 10 mm) semi-preparative column (Phenomenex) with 3 mL/min flow rate with the method: *0-1 min 95% A, 1-15 min 95-40% A, 15-16 min 40-5% A, 16-19 min 5% A, 19-20 min 5-95% A, 20-24 min 95% A*, using detection at 260 nm at room temperature. The buffers used for RP-HPLC were as follows: (A) 50 mM triethylammonium acetate (TEAA), pH ~6.5; (B) 100% acetonitrile. TEAA (1 M) buffer was prepared by dropwise addition of glacial acetic acid (57 ml) to the cooled and stirred mixture of triethylamine (139 ml) in water (800 ml). The pH was adjusted with diluted acetic acid to ~7 and the volume was adjusted to 1 L with water.

Mass spectra were obtained using a HRMS electrospray time-of-flight (ES-TOF) Agilent instrument. Oligonucleotides were eluted using an aqueous mixture of hexafluoroisopropanol and triethylamine with increasing gradient of methanol.

Synthesis

Synthesis of compound 2: 2-(piperazin-1-yl) ethan-1-ol **1** (0.5 g, 3.8 mmol, 1.0 equiv) was dissolved in anhydrous CH_2Cl_2 (30 mL) along with triethylamine (1.080 mL, 7.6 mmol, 2.0 equiv) in a round bottom flask and placed at 0 °C using an ice bath. A solution of commercial benzyloxy carbonyl chloride (600 µL, 0.42 mmol, 1.1 equiv) in anhydrous THF (5 mL) was added by a dropping funnel over 10 min and then allowed to stir at room temperature. After 3-4h, the solvent was removed under reduced pressure and the mixture partitioned between a saturated solution of $NaHCO_3$ (50 mL) and ethyl acetate. The aqueous phase was extracted with ethyl acetate (3x60 mL) and the combined organic layers were washed with brine (15 mL). The organic phase was dried over anhydrous Na_2SO_4, filtered, and concentrated under reduced pressure to afford a thick oil (2.25 g) which was further purified by SiO_2 chromatography (dichloromethane/methanol 25:1 v/v) to give 850 mg (83%) of the product as a pale-yellow oil. Analytical data is matching with the reported data.(1)

Synthesis of compound 3: Compound **2** (0.5 g, 1.8 mmol) was dissolved in CH_2Cl_2 10 mL, then TEA (0.79 mL, 5.6 mmol) followed by *p*-Tosyl chloride (0.46, 2.4 mmol) were added at room temperature. The reaction mixture was stirred at the same temperature for 5-6h and the reaction progress was monitored by TLC. After completion, reaction mixture was diluted with water and extracted with CH_2Cl_2, dried over $MgSO_4$, and concentrated. The crude was used for the next step without further purification.

The above crude compound was dissolved in dry DMF 5 mL and sodium azide (5.0 equiv) was added and reaction mixture was heated at 70 °C overnight. The reaction mixture was cooled down to room temperature and DMF was evaporated. The reaction mixture was then diluted with water and extracted with ethylacetate and dried over $MgSO_4$. Purification by SiO_2 chromatography yielded the compound **3** as a pale-yellow solid (0.32 g, 58%). ^1H NMR (400 MHz, $CDCl_3$) δ 7.40 – 7.30 (m, 5H), 5.15 (s, 2H), 3.55 (t, J = 4.0Hz, 4H), 3.36 (t, J = 2.8 Hz, 2H) 2.62 (t, J = 4.0Hz, 2H). 2.54 -2.42 (m, 4H); ^{13}C NMR (100 MHz, $CDCl_3$) δ 155.19, 136.71, 128.52, 128.05, 127.91, 67.16. 57.21, 52.85, 48.08, 43.74.

Synthesis of compound 4: Compound **3** (150 mg, 0.51 mmol) was dissolved in TFA (3 mL), and the reaction was heated at 80 °C for 12-14 hrs. Reaction completion was monitored through TLC. The reaction mixture was cooled down to room temperature and subjected to evaporation of TFA and washed several times with ether to yield a light yellow solid (93 mg, 71%). ^1H NMR (600 MHz, DMSO) δ 3.59 (t, J = 4.0 Hz, 2H), 3.24 (t, J = 3.6 Hz, 3H), 3.06 - 3.00 (m, 2H), 3.00 - 2.92 (m, 2H); ^{13}C NMR (150 MHz, DMSO) δ, 55.70, 49.16, 46.45, 42.08. HRMS: (*m/z*) calcd for $C_6H_{14}N_5$ $[M+H]^+$: 156.1244 found 156.1248.

Synthesis of compound 6: To the mixture of **5**[1] (100 mg, 0.30 mmol) and amine **4** (TFA salt) (85 mg, 0.33 mmol) in dry DMF (2 mL), triethylamine (100 µL, 0.76 mmol) was added and reaction was heated at 80 °C for 12-14 hrs. The completion of reaction was monitored through LC-MS. On completion, the reaction mixture was allowed to cool, subjected to evaporation of DMF and purified through column chromatography in basic alumina to give a light-yellow compound **6** (90 mg, 65%). ^1H NMR (400 MHz, $CDCl_3$) δ 7.79 (d, J = 8.2 Hz, 1H), 7.30 (d, J = 8.2 Hz), 6.13 (s, 1H), 3.83 (brS, 4H), 3.42 (t, J = 4.8 Hz, 2H), 3.32 (t, J = 7.2Hz, 2H), 3.11 (t, J = 7.2 Hz, 2H), 2.83 (S, 3H), 2.67 (t, J = 6.0Hz, 2H), 2.62 (t, J = 4.8Hz, 4H), 2.37 (S, 3H), 2.24 (t , J = 7.2 Hz, 2H); ^{13}C NMR (100 MHz, $CDCl_3$) δ 169.63, 162.94, 154.62, 150.39, 139.69, 123.80, 121.52, 119.77, 95.34, 57.22, 52.91, 48.24, 43.96, 34.36, 30.49, 24.82, 22.04. HRMS: (*m/z*) calcd for $C_{23}H_{29}N_{10}$ $[M+H]^+$: 445.2571 found 445.2707.

Click conjugation

A 50 µL aliquot of 1 mM oligonucleotide stock was transferred to an Eppendorf to which reagents were added in following order: G4-ligand (compound **6**) (5 equiv. 10 mM stock in DMSO/ACN, 3:7), aqueous DIPEA solution (2.5 µl containing 0.25 µmol, 5 equiv., 0.043 µl) and $CuBr{\cdot}Me_2S$ solution in DMSO (7.5 µl containing 0.5 µmol, 10 equiv., 0.1 mg). The reaction mixture was vortexed and agitated at ambient temperature overnight. The reaction mixture was next diluted with 0.5 mM EDTA (25 µl) solution and water (200 µl) and further purified by RP-HPLC.(2)

Table S2.

The yields and HRMS data of the prepared oligonucleotide conjugates.

No.	Name	Yield (%)	Calculated mass (g/mol)	Found mass HRMS m/z
1.	GL-O15	51	5236.72	5236.08
2.	GL-O16	23	5549.93	5549.13
3.	GL-O17	25	5839.11	5839.17
4.	GL-O15^{ALT}	76	5116.64	5116.64
5.	GL-O15 8T	56	5225.08	5224.98
6.	GL-O15 8&11T	47	5200.08	5200.02
7.	GL-O15^{LNA}	52	5418.82	5418.02
8.	GL-O15 1 MM	49	5245.74	5245.11
9.	GL-O15 2 MM	61	5227.71	5227.08
10.	GL-O15 1,2 MM	47	5236.72	5236.08
11.	GL-O15 14 MM	61	5251.73	5251.11
12.	GL-O15 15 MM	65	5221.71	5221.11
13.	GL-O15 14,15 MM	63	5236.72	5236.08

See mass spectra below.

NMR Spectra of Synthesised Compounds

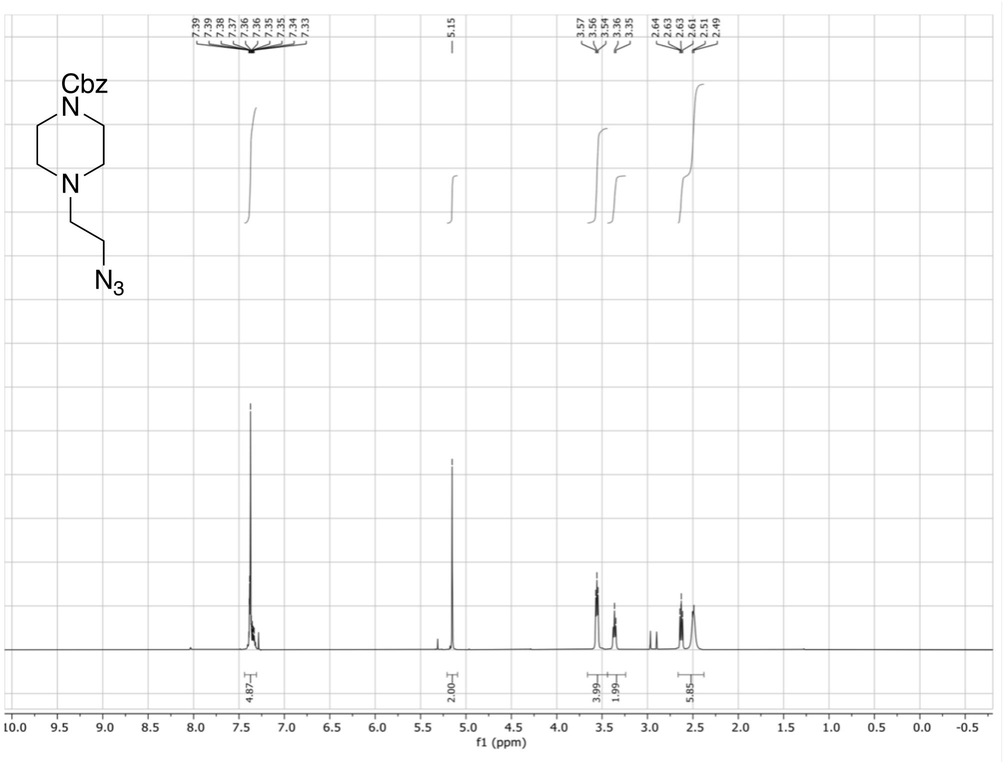

^{1}H NMR spectrum of compound **3** measured in $CDCl_3$ at 400 MHz.

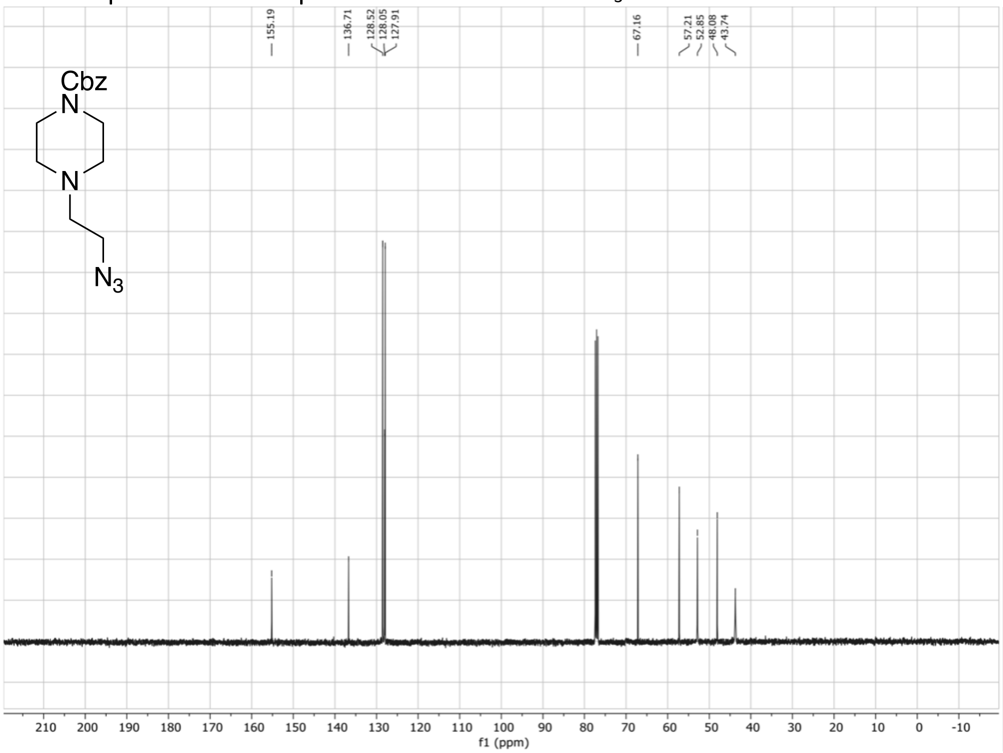

^{13}C NMR spectrum of compound **3** measured in $CDCl_3$ at 125 MHz.

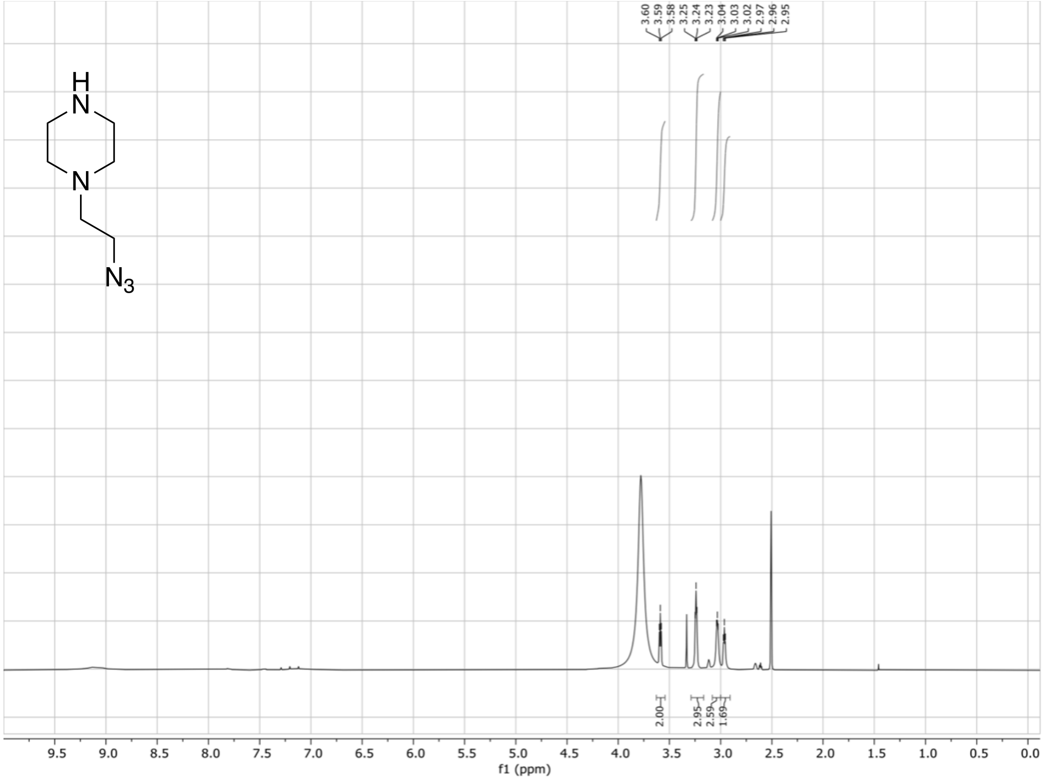

^{1}H NMR spectrum of compound **4** measured in DMSO-d_6 at 400 MHz.

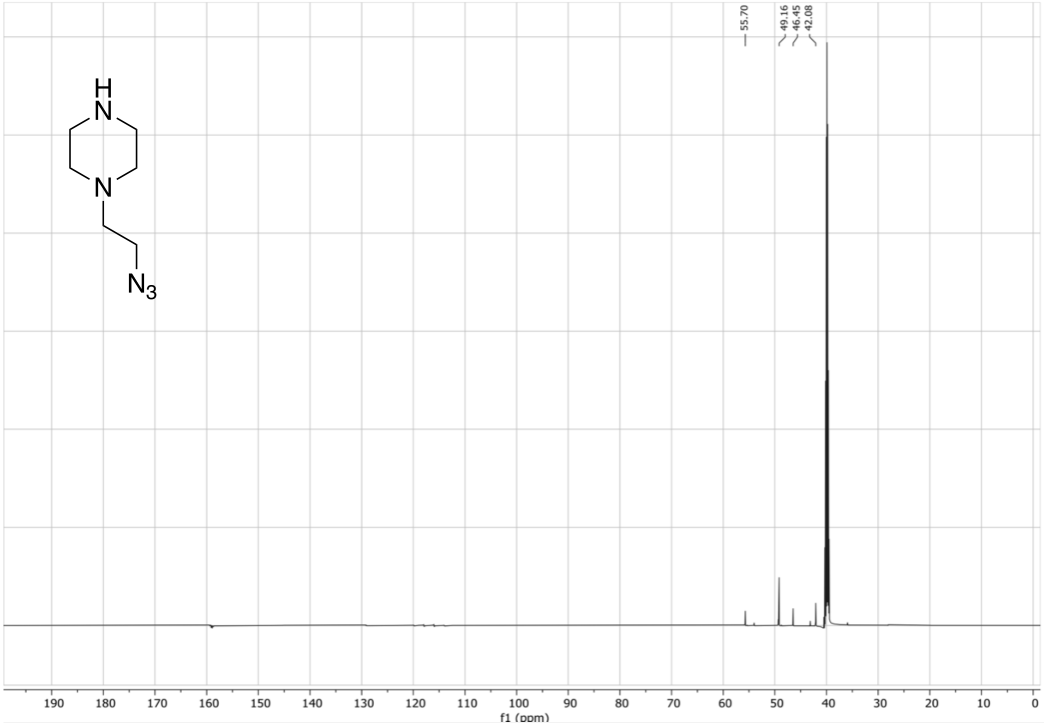

^{13}C NMR spectrum of compound **4** measured in $CDCl_3$ at 125 MHz.

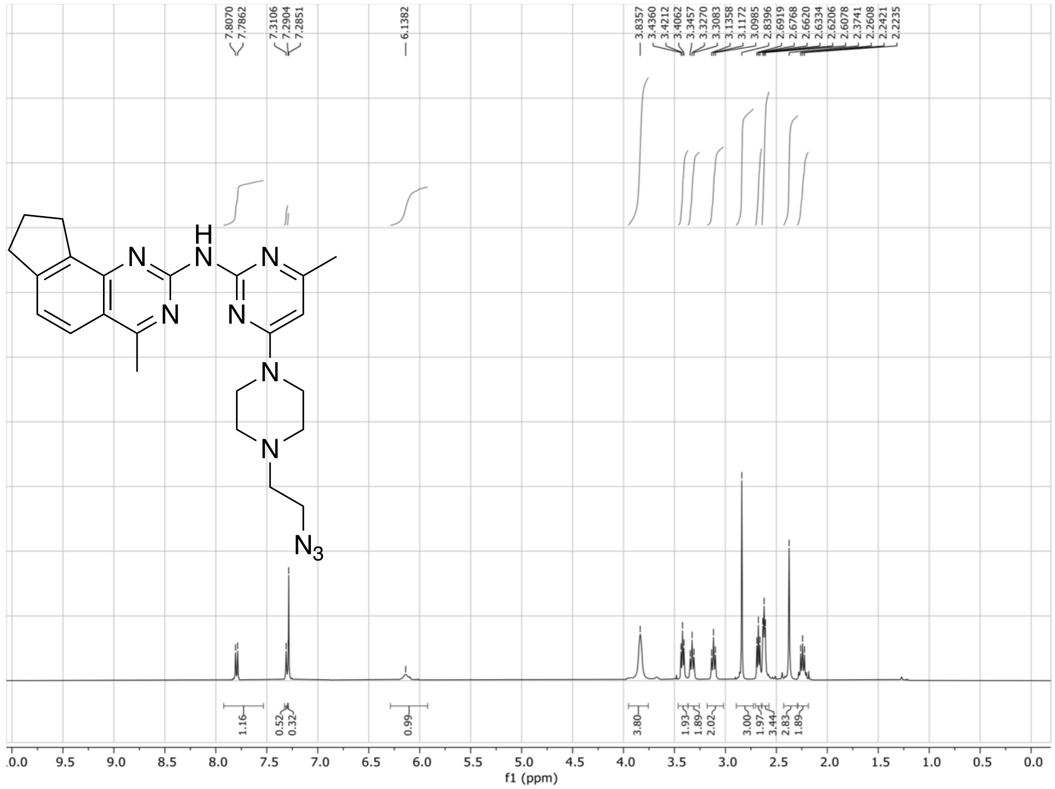

^{1}H NMR spectrum of compound **6** measured in $CDCl_3$ at 400 MHz.

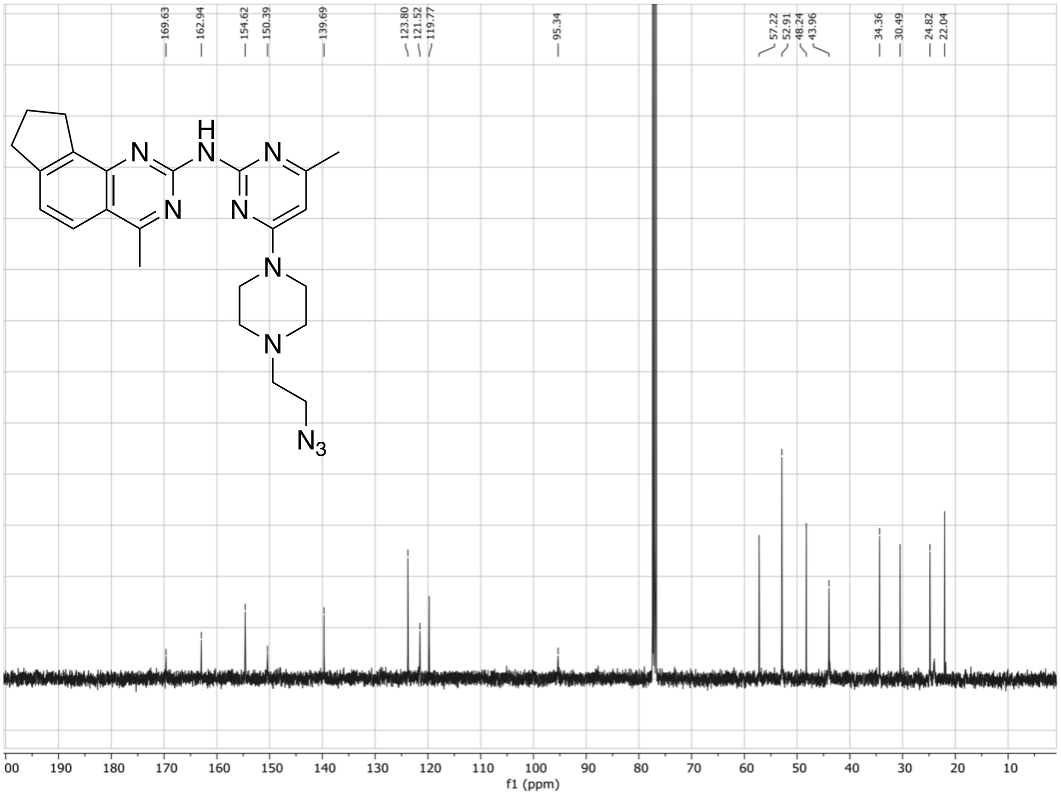

^{13}C NMR spectrum of compound **6** measured in $CDCl_3$ at 125 MHz.

Mass chromatograms of synthesised GL-Os

GL-O15

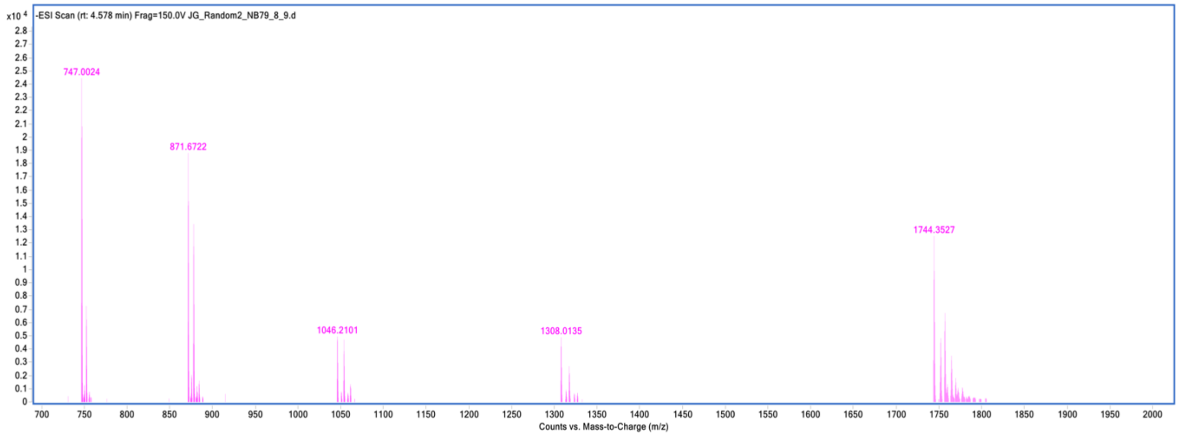

GL-O16

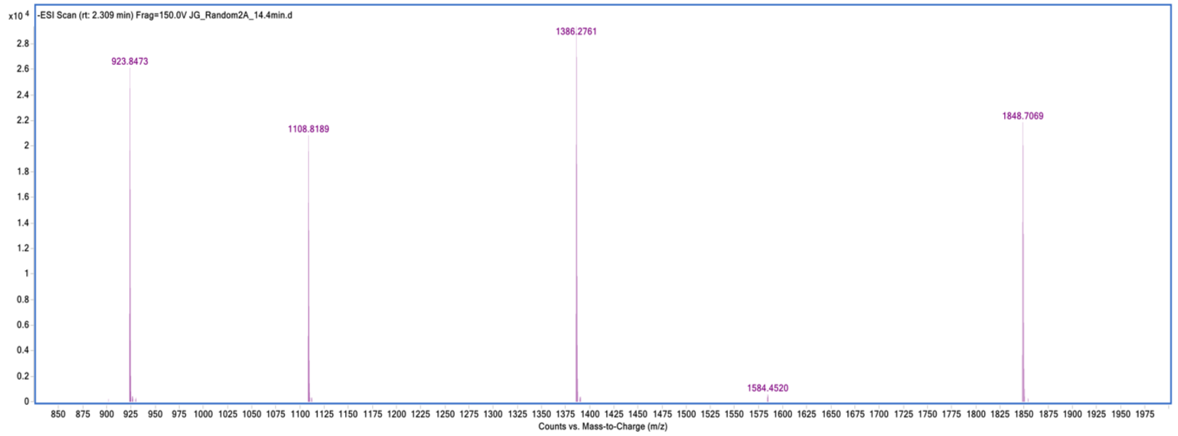

GL-O17

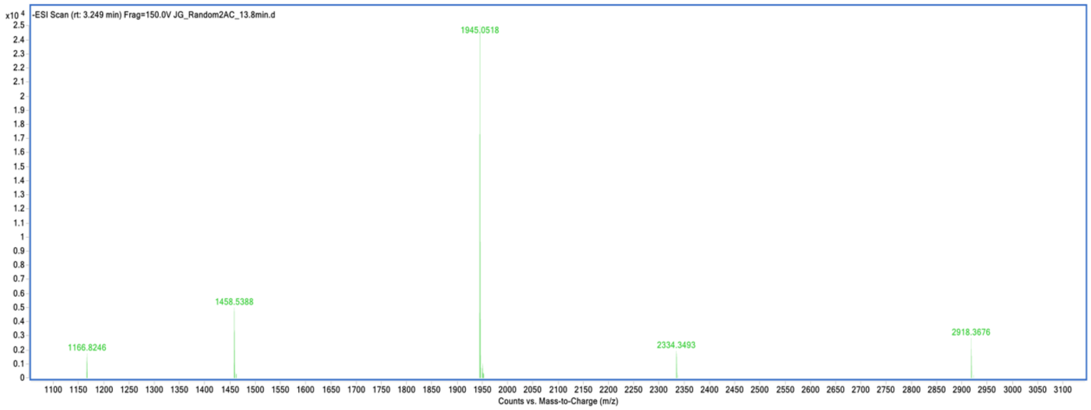

GL-O15^{ALT}

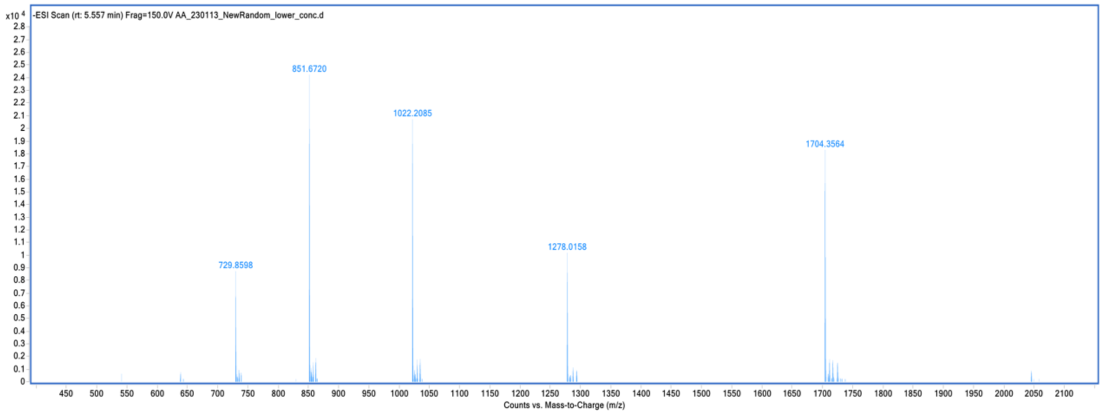

GL-O15 8T

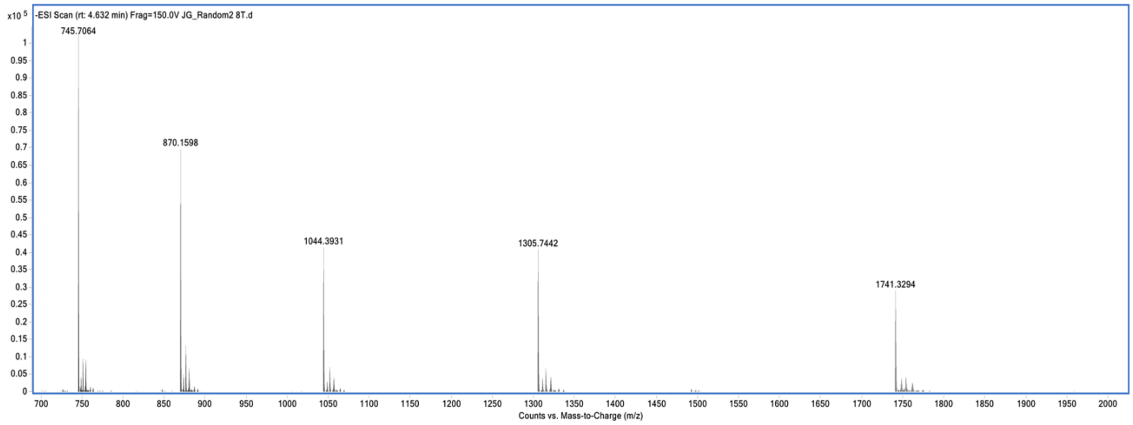

GL-O15 8,11T

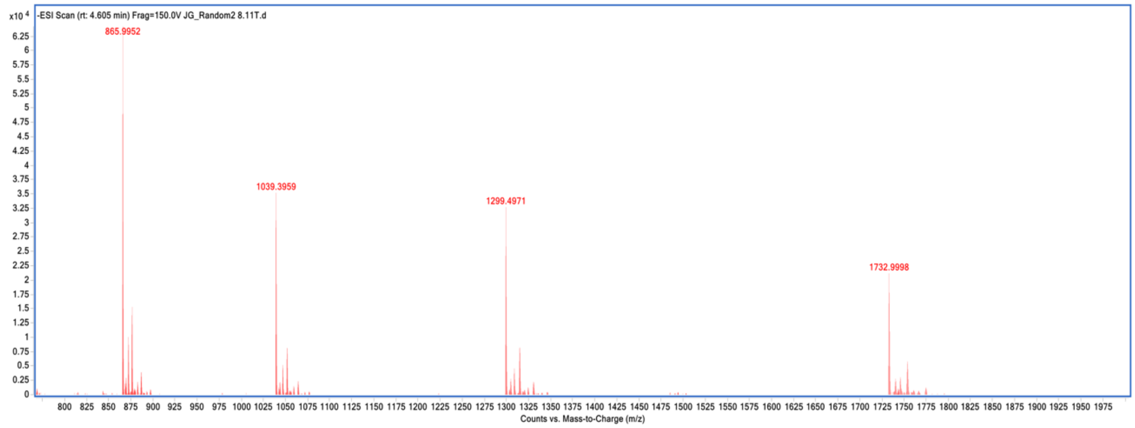

GL-O15 1 MM

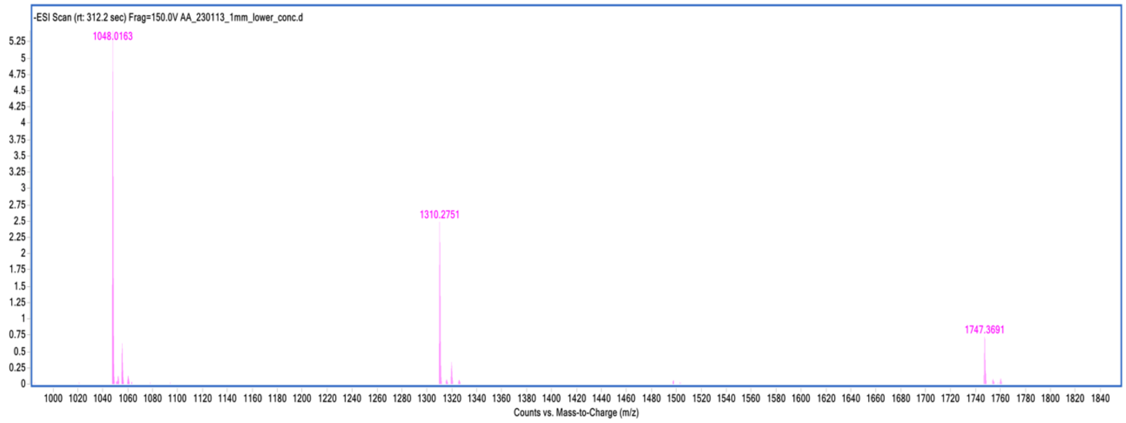

GL-O15 2 MM

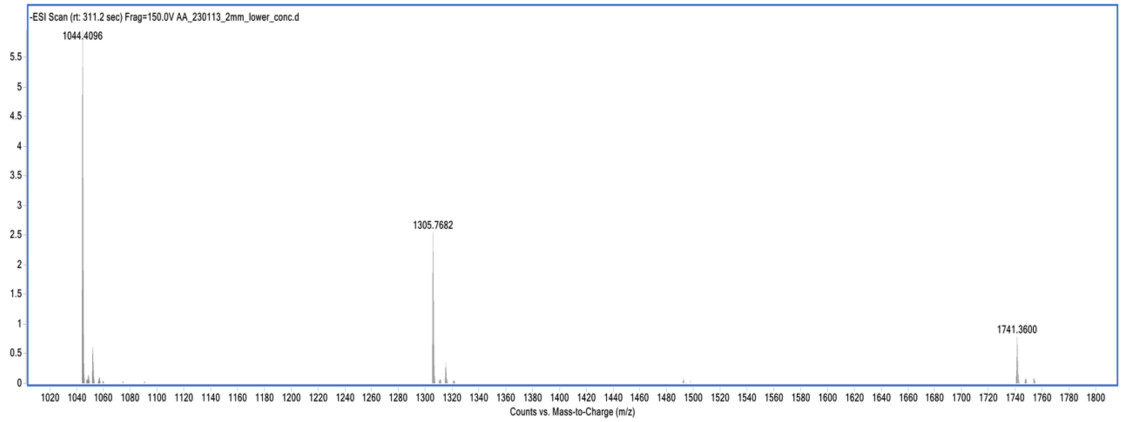

GL-O15 1,2 MM

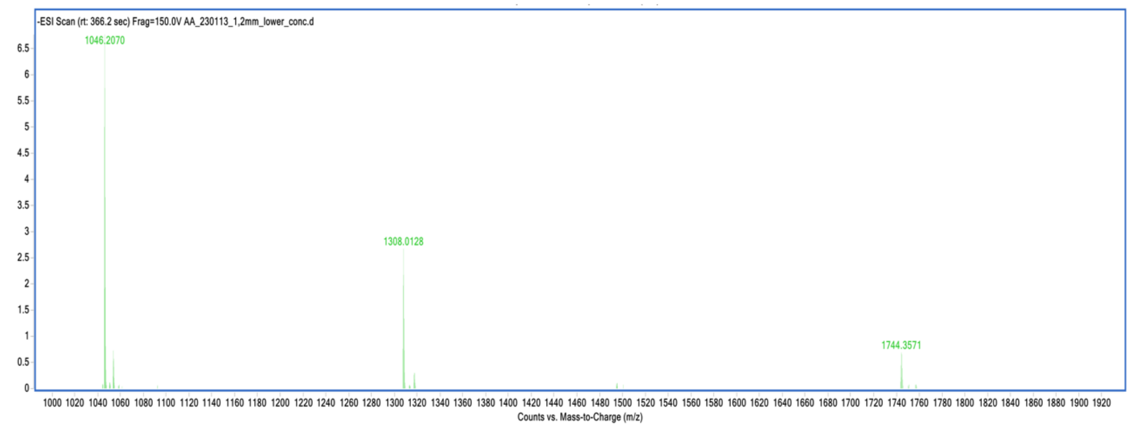

GL-O15 14 MM

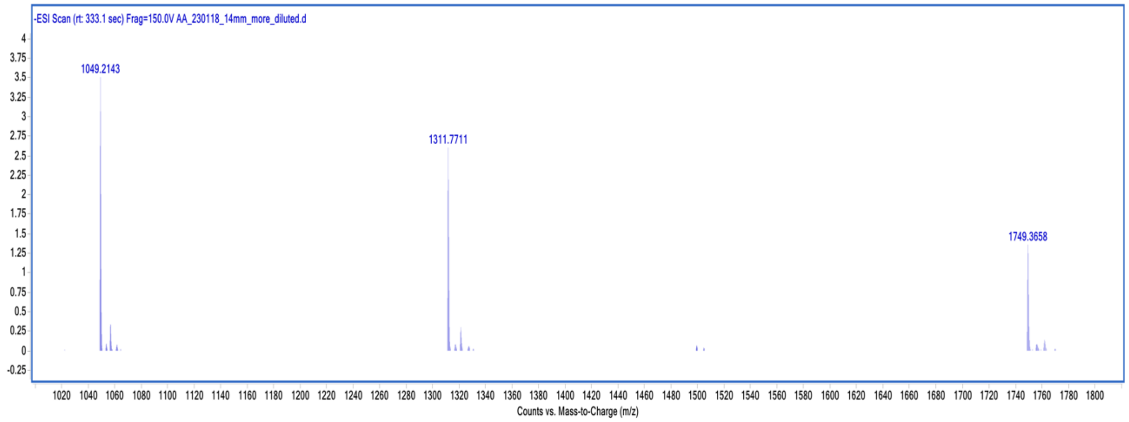

GL-O15 15 MM

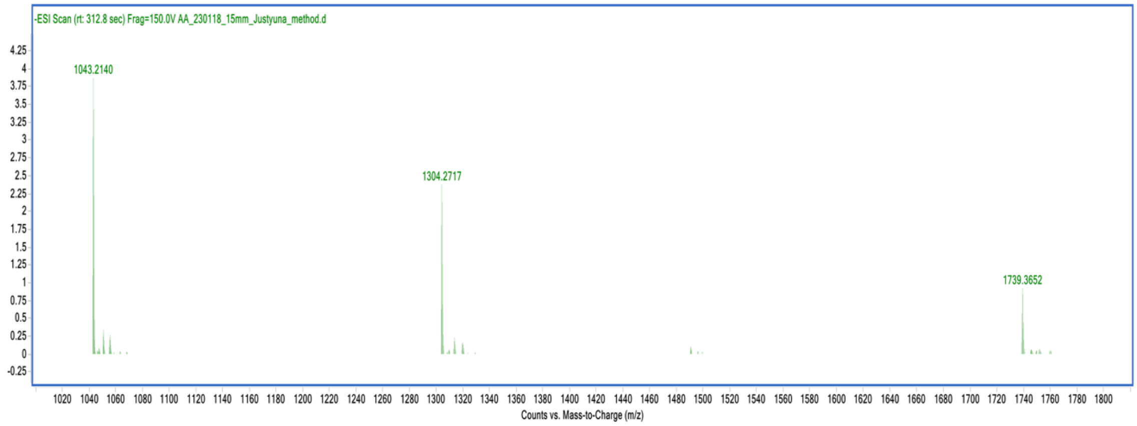

GL-O15 14,15 MM

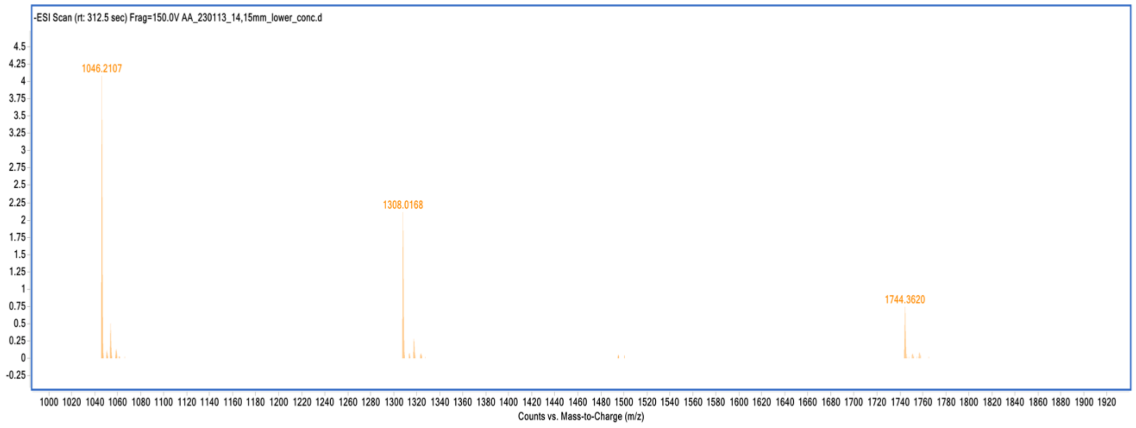

GL-O15^{LNA}

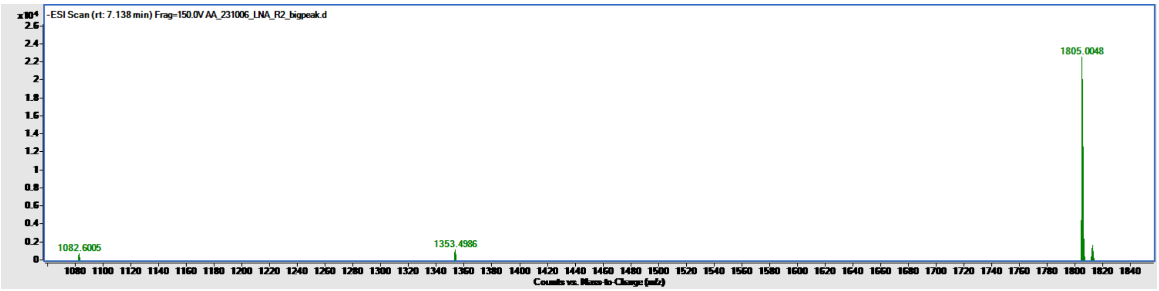

References

1. S. Tomassi *et al.*, From PARP1 to TNKS2 Inhibition: A Structure-Based Approach. *ACS Medicinal Chemistry Letters* **11**, 862-868 (2020).
2. M. Honcharenko, D. Honcharenko, R. Strömberg, Efficient Conjugation to Phosphorothioate Oligonucleotides by Cu-Catalyzed Huisgen 1,3-Dipolar Cycloaddition. *Bioconjugate Chemistry* **30**, 1622-1628 (2019).
